# Ibrutinib in Patients with Relapsed or Refractory Diffuse Large B-Cell Lymphoma: A Retrospective Study

**DOI:** 10.1007/s12288-021-01433-w

**Published:** 2021-04-12

**Authors:** Liangliang Ren, Ling Li, Lei Zhang, Xin Li, Xiaorui Fu, Xinhua Wang, Jingjing Wu, Zhenchang Sun, Xiaoyan Feng, Yu Chang, Zhiyuan Zhou, Feifei Nan, Jiaqin Yan, Fei Kong, Mingzhi Zhang

**Affiliations:** Department of Oncology, Lymphoma Diagnosis and Treatment Center of Henan Province, The First Affiliated Hospital of Zhengzhou University, Zhengzhou, 450052 Henan People’s Republic of China

**Keywords:** Ibrutinib, Relapsed or refractory diffuse large B-cell lymphoma, Efficacy, Safety

## Abstract

Limited treatment options are available for relapsed or refractory diffuse large B cell lymphoma (RR DLBCL). Few clinical studies have reported the use of Ibrutinib, a covalent Bruton Tyrosine kinase (BTK) inhibitor, in RR DLBCL. There are relatively few clinical studies about Ibrutinib in RR DLBCL now. We retrospectively investigated the safety and efficacy of Ibrutinib (alone or in combination with other drugs) in patients with RR DLBCL. We reviewed the medical records of 40 RR DLBCL patients who received Ibrutinib alone or in combination with other drugs in our hospital from June 2018 to August 2020. The objective response rate (ORR) of RR DLBCL patients on Ibrutinib was 22.5%. The median progression free survival time (PFS) was 13.0 months (95% CI 8.914–17.086), and the median overall survival time (OS) was 15.0 months (95% CI 11.931–18.089). Rash (25.0%) and fatigue (25.0%) were the most common adverse reactions in this study. The application of Ibrutinib to patients with RR DLBCL has good short-term efficacy, and the adverse reactions are well tolerated. Combined treatment of Ibrutinib with other drugs has been found to more effective than Ibrutinib therapy alone.

## Introduction

Diffuse large B-cell lymphoma (DLBCL) is the most common type of non-Hodgkin’s lymphoma (NHL) worldwide, accounting for 25% of all cases [1, 2]. Due to heterogeneity in morphology, biological, clinical characteristics and immunophenotype, prognosis of DLBCL varies, resulting in differences in survival rates [3, 4]. Based on gene profiling, DLBCL can be further classified as activated B-cell-like lymphoma (ABC), germinal center B-cell-like lymphoma (GCB) and primary mediastinal B-cell lymphoma [5].

Most patients of DLBCL achieve complete remission (CR) after treatment with R-CHOP (rituximab, cyclophosphamide, doxorubicin, vincristine, prednisone), which is the current standard first-line treatment [6]. The 5-year survival rate after R-CHOP is 50% [7]. One third of DLBCL patients either do not respond to first line treatment or relapse and studies have shown the median survival of these patients to 4–6 months [8, 9]. Patients with RR DLBCL eligible for chemotherapy are salvaged with second line regimens such as ifosfamide, carboplatin, etoposide (ICE); dexamethasone, cytarabine and cisplatin (DHAP), etoposide, cytarabine, cisplatinum and methylprednisolone (ESHAP), gemcitabine, dexamethasone, and cisplatin (GDP). However, the long-term survival rate is less than 10%, and most patients die within 2 years [10]. High-dose chemotherapy plus autologous or allogeneic stem cell transplantation (SCT) remains the standard treatment regimen for young, with non-comorbid RR DLBCL patients [11]. Patients who did not respond to standard chemotherapy or relapsed after SCT are known to have poor prognosis. Anti-CD19 Chimeric antigen receptor T-cell therapy (CAR-T) has shown impressive activity in relapsed or refractory lymphoma, with sustained complete remission for 2 years in RR DLBCL [12]. CAR-T is associated with potentially fatal toxicities, including cytokine release syndrome and neurotoxicity making it ineligible for comorbid or older RR DLBCL with complications [13, 14]. Therefore, treatment of RR DLBCL patients of older age or with comorbid conditions remain challenging [8, 15].

Bruton's Tyrosine kinase inhibitor (BTK) Ibrutinib is highly effective against a variety of B-cell malignancies [16]. Ibrutinib is a potent, irreversible and effective inhibitor of BTK that is taken orally, and was approved for indications in mantle cell lymphoma (MCL) and chronic lymphocytic leukemia (CLL) in 2013 and 2014 [17–19].

At present, there are relatively few clinical research about Ibrutinib in RR DLBCL. Therefore, this study used a retrospective study to evaluate the efficacy and safety of Ibrutinib in 40 patients with RR DLBCL.

## Material and Methods

**Fig. 1:**
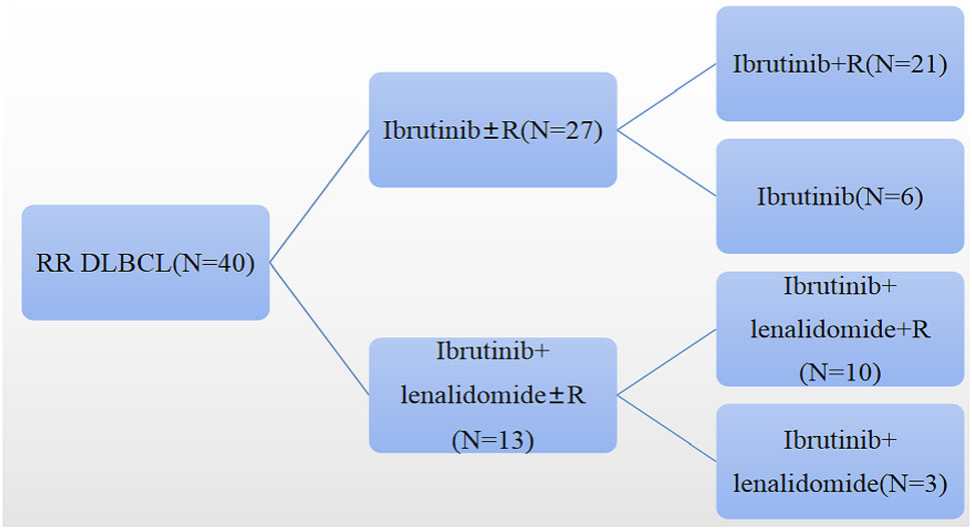
Specific drug regimen of 40 patients with relapsed or refractory Diffuse Large B-cell Lymphoma

We reviewed 40 patients with RR DLBCL who were treated with Ibrutinib (or combined with other drugs) in the First Affiliated Hospital of Zhengzhou University from June 2018 to August 2020(median age, 61 years; median follow up, 11.5 months). The 40 patients were divided into the Ibrutinib ± R group and Ibrutinib + lenalidomide ± R group. To details about the Ibrutinib regimen given to RR DLBCL patients is shown in Fig. 1 and the baseline characteristics is shown in Table 1.

**Table 1:** Baseline characteristics

Clinical features	Ibrutinib ± R (%) (n = 27)	Ibrutinib + lenalidomide ± R (%) (n = 13)
The general informations		
Gender		
Male	15 (55.6)	7 (53.8)
Female	12 (44.4)	6 (46.2)
Age (year)		
> 60	14 (51.9)	7 (53.8)
≤ 60	13 (48.1)	6 (46.2)
ECOG		
< 2	13 (48.1)	8 (61.5)
≥ 2	14 (51.9)	5 (38.5)
Pathological characteristics		
EBER		
Negative	23 (85.2)	12 (92.3)
Positive	4 (14.8)	1 (7.7)
Ki-67		
< 50%	1 (3.7)	2 (15.4)
≥ 50%	26 (96.3)	11 (84.6)
COO subtype		
GCB	1 (3.7)	2 (15.4)
Non-GCB	26 (96.3)	11 (84.6)
Ann Arbor stage		
I–II	3 (11.1)	2 (15.4)
III–IV	24 (88.9)	11 (84.6)
Previous chemotherapy		
≤ 2	7 (26.0)	3 (23.1)
> 2	20 (74.0)	10 (76.9)
IPI score		
≤ 3	14 (51.9)	11 (84.6)
> 3	13 (48.1)	2 (15.4)
Extranodal sites		
≤ 1	10 (37.0)	6 (46.2)
> 1	17 (63.0)	7 (53.8)
Laboratory examinations		
LDH		
Normal	9 (33.3)	8 (61.5)
Increased	18 (66.7)	5 (38.5)
Serum β2 microglobulin		
Normal	22 (81.5)	5 (38.5)
Increased	5 (18.5)	8 (61.5)
Disease characteristics		
B symptoms		
Absent	19 (70.4)	6 (46.2)
Present	8 (29.6)	7 (53.8)

*COO* cell of origin, *GCB* germinal centre B-cell, *IPI* international prognostic index, *LDH* lactate dehydrogenase

### Inclusion and Exclusion Criteria

Inclusion criteria: The histopathological type of the primary lesions was confirmed as DLBCL by two senior pathologists; Failure or relapse after treatment with standard second-line or above; Patients with at least one measurable lesion; Age over 18 years old; Have not previously received BTK inhibitor treatment. Exclusion criteria: Pregnant and lactating women; Accompanied by severe organ damage or failure.

### Study Design

This study aimed to evaluate the effectiveness and safety of Ibrutinib in the treatment of 40 patients with RR DLBCL. All patients recieved Ibrutinib orally for more than one month. They received Ibrutinib orally, 140 mg × 4 tablets, a total dose of 560 mg one day, until disease progression or death. In case of intolerance to Ibrutinib, either the dose was reduced to 420 mg per day or was withheld for a maximum of one week. Treatment with Ibrutinib for 21 days was considered as one cycle of treatment and efficacy evaluation was performed after every 2 cycles of treatment. Efficacy assessment was done on the basis of physical examination, Eastern Cooperative Oncology Group (ECOG) status assessment, bone marrow aspiration and biopsy (if bone marrow involved), radiologic examination using color Doppler ultrasonography and enhanced computed tomography (CT) or positron emission tomography-computed tomography (PET-CT) of involved organs.

We retrospectively evaluated efficacy and safety of Ibrutinib in 40 patients with RR DLBCL. Twenty-seven patients received Ibrutinib ± R regimen. Rituximab was added with Ibrutinib in patients who have received 8 cycles of rituximab during previous lines of treatment. There are thirteen patients recieved Ibrutinib + lenalidomide ± R. They received 10 mg of lenalidomide orally every day, and lenalidomide was taken for 2 weeks and stopped for 1 week. The application principles of Ibrutinib and rituximab are also stated above.

### Assessments

Twenty-one days is one treatment cycle. Efficacy is evaluated every 2 cycles. According to WHO criteria, it is divided into: complete remission (CR); partial remission (PR), stable disease (SD), and progressive disease (PD). Objective response rate (ORR) = (CR + PR)/(CR + PR + SD + PD) × 100%, refers to the proportion of patients whose tumors have shrunk to a certain amount and maintained for a certain period of time. CR is defined as complete disappearance of the lesion. PR is defined as a reduction in lesion volume of more than half and no new lesions. PD is defined as lesions that has increased in volume by more than 25% or are new. SD is between PD and PR.

The long-term efficacy assessment indexes are: progression-free survival (PFS) and overall survival (OS). PFS refers to the time from the first administration of Ibrutinib until disease progression or death. OS is the time from the first administration of Ibrutinib to death from any cause or the last follow-up.

SPSS 24.0 statistical software was used for data processing, analysis and relevant statistical chart drawing. Kaplan–Meier method was used to draw the survival curve, and Cox proportional hazard regression model was used to analyze the factors affecting PFS and OS of patients with RR DLBCL. *P* < 0.05 indicates that the difference is statistically significant.

## Results

### Short-Term Efficacy

**Fig. 2:**
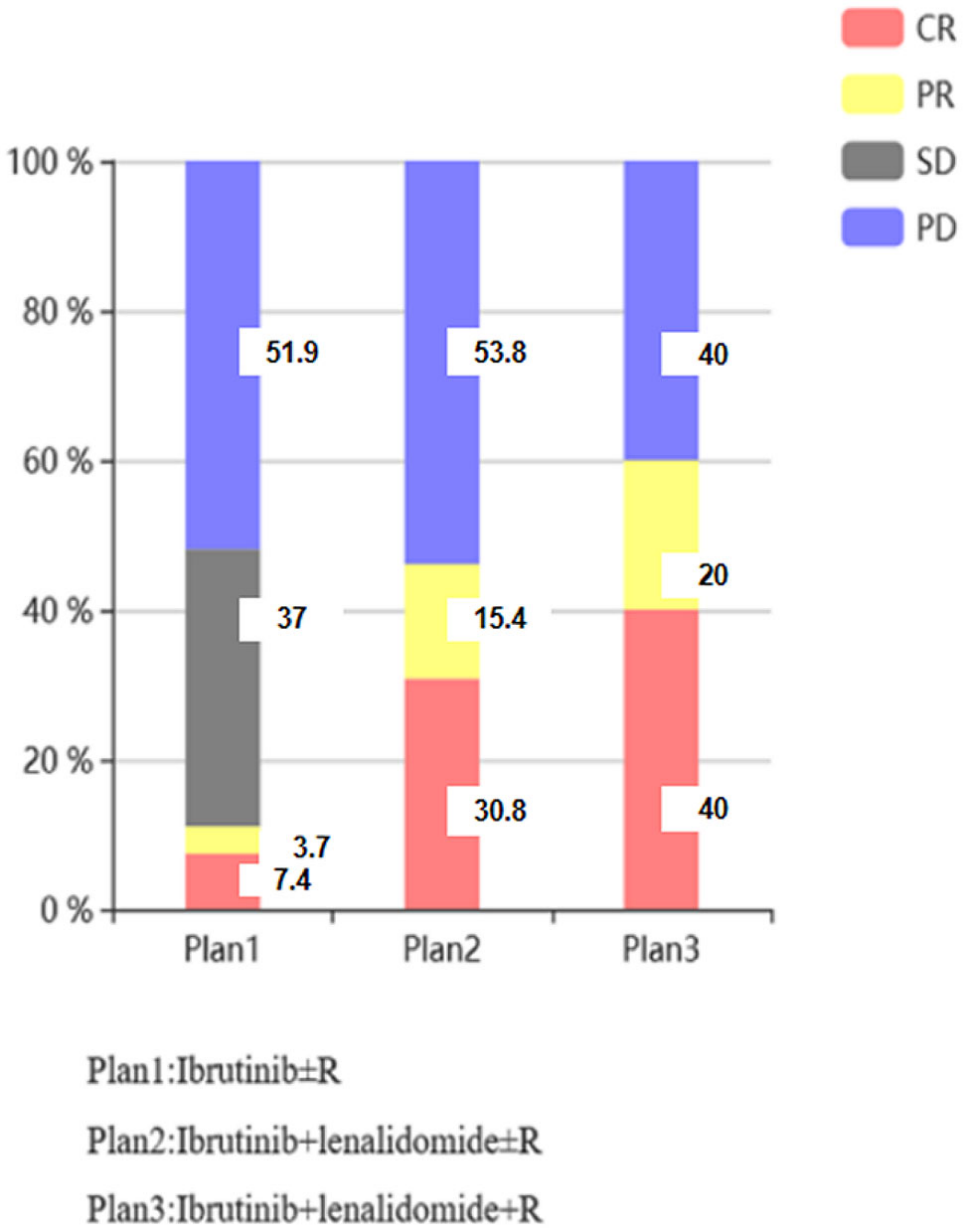
A stacked column of efficacy evaluation

The data collection cut-off time for the primary analysis was August 2020. Six patients (15.0%) had achieved CR, with three cases of PR (7.5%) and ten cases (25.0%) of SD. Nine of the forty (22.5%) patients had achieved ORR. In particular, ten of them were in Ibrutinib + lenalidomide + R group, CR was 40%, PR was 20%. Its ORR reached 60.0%. The specific follow-up results are shown in Fig. 2.

### Long-Term Efficacy

**Fig. 3:**
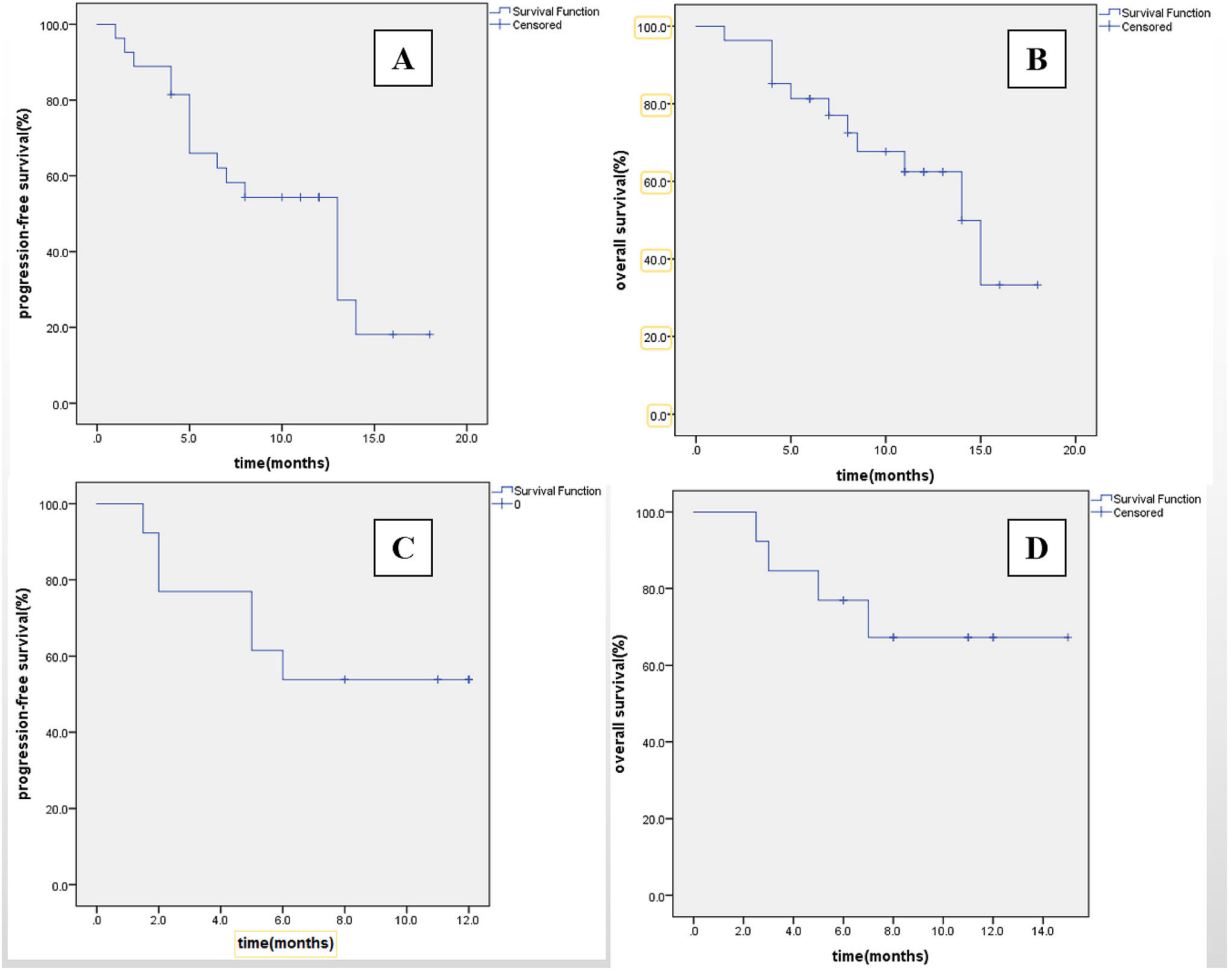
A Ibrutinib ± R, Kaplan–Meier graph for progression-free survival (n = 27); B Ibrutinib ± R, Kaplan–Meier graph for overall survival (n = 27); C Ibrutinib + lenalidomide ± R, Kaplan–Meier graph for progression-free survival (n = 13); D Ibrutinib + lenalidomide ± R, Kaplan–Meier graph for overall survival (n = 13)

For 40 patients with RR DLBCL, the median PFS was 13 months (95% CI 8.914–17.086) and the median OS was 15 months (95% CI 11.931–18.069). In Ibrutinib ± R group, the median PFS was 13 months (95% CI 8.411–17.589) and the median OS was 14 months (95% CI 10.286–17.714). The median PFS and the median OS in Ibrutinib + lenalidomide ± R group did not reach. Kaplan–Meier estimates graph of PFS and OS are shown in Fig. 3. Three patients in our cohort did not respond to multiple lines of treatment and were considered as primary refractory patients. These patients did not respond to Ibrutinib ± chemotherapy. The short-term efficacy and long-term efficacy results of the 3 patients are shown in Table 2.

**Table 2:** Data of primary drug-resistant patients

Patient ID	Short-term efficacy	Long-term efficacy		Regimens
		PFS (months)	OS (months)	
1	PD	5	6	Ibrutinib + R
2	PD	1.5	2.5	Ibrutinib + R + lenalidomide
3	PD	5	6	Ibrutinib + R

### Assessment of Adverse Events

In this study, Common Terminology Criteria for Adverse Events Version 5.0 (CTCAE Version 5.0) was used to assess adverse events, which was used by the US National Institutes of Health in 2017 [20, 21]. The grade of adverse events was 1–5. No Grade 5 appeared in this study (Grade 5 means death).

We observed mild adverse events (Grade 1–2). In Ibrutinib ± R group, rash (8 patients, 29.6%) and inappetence (6 patients, 22.2%) were the most common adverse events. And in Ibrutinib + lenalidomide ± R group, the most common adverse events were nausea (4 patients, 30.8%) and fatigue (3 patients, 23.1%). In Ibrutinib ± R group, one patient developed severe urine tract bleeding for which Ibrutinib was discontinued and managed with haemostatic drugs and blood products. After 3 days, bleeding was controlled and the patient remained under observation for next 2 days. Under supervision, the patient was rechallenged with Ibrutinib and it was well tolerated thereafter. Two patients experienced severe fatigue, which was managed by reducing dose of Ibrutinib to 420 mg/day. Major treatment related adverse events for each treatment group were reported in Table 3. None of the patient developed hypertension.

**Table 3:** Major treatment-related adverse events

Adverse events	Ibrutinib ± R (%)		Ibrutinib + lenalidomide ± R (%)	
	Grade 1/2	Grade 3/4	Grade 1/2	Grade 3/4
Fatigue	5 (18.5)	1 (3.7)	3 (23.1)	1 (11.1)
Nausea	4 (14.8)	0	4 (30.8)	0
Vomiting	3 (11.1)	0	1 (7.7)	0
Inappetence	6 (22.2)	0	2 (15.4)	0
Diarrhea	4 (14.8)	0	2 (15.4)	0
Rash	8 (29.6)	1 (3.7)	1 (7.7)	0
Anemia	3 (11.1)	0	2 (15.4)	0
Neutropenia	5 (18.5)	0	2 (15.4)	0
Hemorrhage	2 (7.4)	1 (3.7)	0	0
Atrial fibrillllatation	3 (11.1)	0	1 (7..7)	0
Respiratory tract infection	1 (3.7)	0	0	0
Arthralgia	1 (3.7)	0	1 (7.7)	0
Cough	1 (3.7)	0	0	0

### Analysis of Prognostic Factors

Cox proportional hazard regression model analyzed the factors affecting PFS and OS in 40 patients in this study. The results showed that ECOG score and COO subtype were significantly correlated with PFS and OS (HR and *P* value are in Tables 4 and 5). Patients with ECOG PS < 2 or non-GCB DLBCL were found to have better PFS and OS. *P* value < 0.05 was considered statistically significant.

**Table 4:** Analysis of prognostic factors affecting PFS

Various	Univariate analysis		Multivariate analysis	
	HR (95% CI)	*P*	HR (95% CI)	*P*
Gender	0737 (0.316–1.717)	0.480	–	–
age	0.473 (0.203–1.103)	0.083	–	–
ECOG	4.516 (1.522–13.400)	0.007	4.213 (1.410–12.585)	0.010
Ki-67	1.515 (0.201–11.394)	0.687	–	–
COO subtype	0.172 (0.047–0.626)	0.008	0.221 (0.061–0.803)	0.022
Ann Arbor stage	5.418 (0.728–40.337)	0.099	–	–
Previous chemotherapy	3.515 (0.820–15.060)	0.090	–	
IPI score	0.913 (0.387–2.153)	0.835	–	–
Extranodal sites	1.401 (0.590–3.329)	0.445	–	–
LDH	0.472 (0.190–1.176)	0.107	–	
β2 microglobulin	0.679 (0.230–2.007)	0.484	–	–
B symptoms	0.899 (0.353–2.287)	0.822	–	–

**Table 5:** Analysis of prognostic factors affecting OS

Various	Univariate analysis		Multivariate analysis	
	HR (95% CI)	*P*	HR (95% CI)	*P*
Gender	0795 (0.295–2.139)	0.650	–	–
Age	0.390 (0.132–1.147)	0.087	–	–
ECOG	4.149 (1.178–14.608)	0.027	3.883 (1.101–13.694)	0.035
Ki-67	1.041 (0.135–8.027)	0.969	–	–
COO subtype	0.153 (0.040–0.581)	0.006	0.175 (0.046–0.065)	0.010
Ann Arbor stage	3.700 (0.487–28.109)	0.206	–	–
Previous chemotherapy	2.265 (0.514–9.988)	0.280	–	
IPI score	1.322 (0.487–3.585)	0.584	–	–
Extranodal sites	0.915 (0.339–2.469)	0.861	–	–
LDH	0.581 (0.217–1.560)	0.281	–	
β2 microglobulin	0.484 (0.110–2.138)	0.338	–	–
B symptoms	1.246 (0.444–3.500)	0.676	–	–

## Discussion

In recent years, Ibrutinib has been found to have strong antitumor activity in different B-cell malignancies [22]. Kelu Hou [7] conducted a meta-analysis of Ibrutinib therapy and reported overall response and CR as 49.7% and 27.7% respectively in RR DLBCL. Studies have shown that when Ibrutinib is combined with lenalidomide, the activity of DLBCL model increases, especially in non-germinal center B-cell-like (non-GCB) even in ABC DLBCL, which is consistent with BCR and MYD 88 signaling pathway [23]. It is suggested that clinical efficacy usually benefits from combination therapy compared with monotherapy. In addition, molecular analysis showed that patients with both MYD88 and CD79B mutations were the most sensitive to Ibrutinib [24]. Because of the short duration of this study and the limited number of specimens, genetic testing was not performed on it. This study will be gradually improved in subsequent studies. In this study, only 6 patients received Ibrutinib alone, including 1 patient achieved CR (15.0%), 4 patients achieved SD (10.0%) and 1 patient achieved PD (15.0%). This result makes us think about the resistance of Ibrutinib. In clinical practice, intrinsic and acquired resistance to Ibrutinib has indeed been observed.

The main adverse reactions in this study were grade 1–2, and grade 3–4 were relatively rare. Common adverse reactions were tolerable. For rare grade 3–4 adverse reactions, they could be recovered after stopping the drug for less than 1 week and symptomatic treatment was actively conducted. Paydas [25] mentioned that in early clinical trials, BTK inhibitors have been reported to be associated with increased rates of bleeding, including subdural hematoma, gastrointestinal bleeding and hematuria. However, the effect of BTK inhibitors on platelet function and bleeding risk is unclear. On the one hand, the dosage of Ibrutinib can be reduced. On the other hand, the indications and dosage of antithrombotic drugs are also important, and platelet transfusion should be considered even if there is no thrombocytopenia in the case of severe bleeding [26]. Salem [27] noted that Ibrutinib can induce cardiovascular events, and cardiovascular toxicity is the leading cause of death. He found that Ibrutinib was associated with supraventricular arrhythmias (atrial fibrillation is the most common form), heart failure, ventricular arrhythmias, and conduction disorders. Brown [28] analyzed the data of 1505 patients treated with Ibrutinib and found that the previous history of atrial fibrillation and the age more than 65 years olds were independent risk factors for the occurrence of atrial fibrillation. In the end, it was concluded that atrial fibrillation occurred during the application of Ibrutinib is usually controllable without stopping the drug. Fortunately, Ibrutinib-induced arrhythmias (especially atrial fibrillation) were less likely to occur in this study and may be related to drug interactions and drug dosage. Tilly Varughese [29] analyzed the risk of severe infection in patients with lymphoma treated with Ibrutinib. For patients with lymphoma treated with Ibrutinib, he proposed that aggressive fungal infections were related to the use of corticosteroids at any time and to receive 3 previous anti-tumor regimens. The only factor associated with bacterial infection was the reduction of neutrophils. Therefore, the situation of infection should be closely monitored during the medication and targeted preventive measures should be taken actively. As the oral dose of Ibrutinib increases, the risk of hypertension will increase, and the incidence of grade III hypertension may be as high as 25% [25]. Seongseok Yun [30] proposed that patients with a tendency to worsen hypertension during treatment with Ibrutinib may also have an increased risk of atrial fibrillation. No patients with hypertension were found in this study. It may be related to the fact that 40 patients did not have hypertension before. Blood pressure and heart rate should be detected before and after the application of Ibrutinib, and antihypertensive drugs should be given in time. Diarrhea is a common side effect of Ibrutinib treatment, but its severity is not high, and it is a self-limited disease. It most often occurs in the first six months of treatment, with a median duration of 20 days [25].

Among the 40 patients in this study, CR was 15.0%, PR was 7.5%, SD was 25.0%, 9 patients achieved ORR (22.5%). But for patients with RR DLBCL: (1) About 30% of DLBCL patients will have recurrence, and the prognosis of patients who relapse after first-line treatment is extremely poor [31]; (2) The treatment options for refractory DLBCL are limited. In this study, CR and PR were lower after the treatment with Ibrutinib. But the long-term stability of RR DLBCL could be regarded as another therapeutic direction, and the short-term efficacy was acceptable. It also shows that our current plan still has defects.

In 2019, Andre Goy [32] initiated a clinical trial related to "Ibrutinib + lenalidomide + R in RR DLBCL". The ORR of non-GCB DLBCL patients was 65% (CR: 41%). The result indicates that Ibrutinib + lenalidomide + R showed better activity and controllable safety in patients with RR DLBCL (especially non-GCB DLBCL). Surprisingly, in our retrospective study, there were 10 patients with Ibrutinib + lenalidomide + R regimen, and their ORR reached 60.0%. There were 7 patients with non-GCB DLBCL and the ORR achieved 71.4% (3 patients achieved CR;2 patients achieved PR). For the Ibrutinib + lenalidomide + R regimen, relevant reports should continue to be collected, the number of cases should be appropriately expanded to reduce the experimental error. Although the number of cases in Ibrutinib + lenalidomide ± R group was small, the median PFS and OS were not reached during the follow-up period, indicating that the long-term treatment effect may be better than Ibrutinib ± R group. In the later period, we will expand the number of cases in the Ibrutinib + lenalidomide ± R group, extend the follow-up time, and observe the efficacy.

Michael Crump [33] mentioned that the results of the study of patients with refractory DLBCL after failure of second-line treatment or patients who relapsed after ASCT showed that their clinical prognosis was poor (The median OS was 5 months and 8–10 months, respectively). For patients with refractory DLBCL, the ORR to the next-line treatment was 26% (The CR rate was 7%), and the median OS was 6.3 months. The results of this study are better than this one. Programmed death receptor-1 antibodies, tyrosine kinase inhibitors, anti-CD79B antibody–drug conjugates, PIK3 inhibitors, and BCL-2 inhibitors are all used in the treatment of RR DLBCL. These single drugs have multiple ORRs. The ORR of these single drugs is mostly between 10–60% [10].

Compared with other treatment regimens for RR DLBCL, ORR in the Ibrutinib ± R group did not achieve the expected effect, while Ibrutinib + Lenalidomide ± R group showed better efficacy. Again, it was shown that the combined treatment of Ibrutinib was more effective than the single treatment.

## Conclusion

Our study showed that the application of Ibrutinib to patients with RR DLBCL has good short-term efficacy, and the adverse reactions can be tolerated. Combined therapy was more effective than single therapy. Therefore, the appropriate drug combination therapy should be selected on the premise of paying attention to the side effects. In the near future, whether Ibrutinib combined with other drugs can play a greater potential in the treatment of RR DLBCL deserves more in-depth study in the future.

## Declarations

### Availability of Data and Material (Data Transparency)

All data and materials are true and valid. All tables and figures are original.

### Code Availability (Software Application or Custom Code)

SPSS 24.0 statistical software.
